# Percutaneous radiofrequency ablation of ovarian cancer metastasis in the spleen: a therapeutic option to consider

**DOI:** 10.1007/s40477-024-00921-9

**Published:** 2024-06-20

**Authors:** Andrea Boccatonda, Paula Antonia Mauloni, Monica Cevenini, Livia Masi, Sofia Maria Bakken, Carla Serra

**Affiliations:** 1 Department of Medical and Surgical Sciences, University of Bologna, Bologna, Italy; 2 Interventional Diagnostic and Therapeutical Ultrasound Unit, Department of Organ Failure and Transplantation S, Orsola-Malpighi Hospital-University of Bologna, Via Massarenti N 9, 40138 Bologna, BO Italy

**Keywords:** Radiofrequency ablation, Thermal ablation, Metastasis, Ovarian cancer, Spleen

## Abstract

Splenic metastasis are rare clinical entities developing in less than 1% of all metastatic cancers and usually in the setting of disseminated disease. To date, splenectomy is traditionally the first line therapy in patient with splenic metastasis, however non-surgical therapies have been reported. Here we described the case of a 57-year-old patient with splenic metastasis from ovarian cancer successfully treated by percutaneous radiofrequency ablation. Furthermore, we performed a literature systematic review of the cases of splenic metastases treated by thermal ablation.

## Introduction

In solid organ tumors, the splenic localization of a metastasis is rare and represents less than 1% of all types of metastasis. Breast, lung, colorectal and ovarian cancers and melanoma represent the most common primary origins for splenic metastasis and splenic involvement frequently characterizes the late stages of a multi-visceral metastatic process [1]. Nowadays, the treatment of splenic metastasis has been reserved to isolated ones, and the splenectomy is the first-line choice for treating an isolated splenic metastasis. However, in last years, the literature reported that 25–50% of the spleen parenchyma has to be preserved in order to prevent serious infectious. Indeed, several data have shown that the death rate from all type of infections after splenectomy is up to 600 times greater than in the general population. Therefore, the setup of safe and effective minimally invasive techniques to treat spleen focal lesions is needed [2].

Recently, thermal ablation (TA) techniques found a wide application in the treatment of solid tumors of various organs, in particular the liver and the kidneys [3].

The TA of the spleen has not been widely described; the treatment of cirrhosis and thalassemia related hypersplenism and hemostasis in trauma and in partial splenectomies are its main indications [4]. Few data about local control of splenic metastatic lesions have also been reported [5]. Here we report the case of a patient with spleen metastasis from ovarian carcinoma treated with percutaneous radiofrequency ablation (RFA).

## Case report

A 57-year-old woman was referred to our Ultrasound Unit for imaging follow-up for ovarian cancer diagnosed in 2013. She had no significant disease except the ovarian cancer, and she was not on any medication. As first line treatment, she underwent a hysterectomy and bilateral salpingo-oophorectomy with omentectomy and lymphadenectomy. Adjuvant chemotherapy with taxol/carboplatin was performed. In January 2015, the patient underwent laparotomic adhesiolysis, removal of the meso-rectal metastasis, wedge resection in VII liver segment, right diaphragmatic peritonectomy and hyperthermic intraperitoneal chemotherapy due to disease progression. In March 2018 a FDG positron emission tomography/computed tomography (PET/CT) scan showed two new uptake area in the spleen, near the hilum. No other lesions were found in the other solid organs. A multidisciplinary evaluation with the Oncologists was performed, and a percutaneous RFA treatment was preferred instead of additional surgical procedures due to the surgical and infectious risk. At the time of the evaluation, the spleen was the only site with active neoplastic disease; furthermore, RFA was considered less invasive than splenectomy, by considering the high risk of recurrence.

Since the spleen lesion was near the ilum, RF technique was chosen to decrease the risk of vessel damage in comparison with microwave technique.

**Fig. 1:**
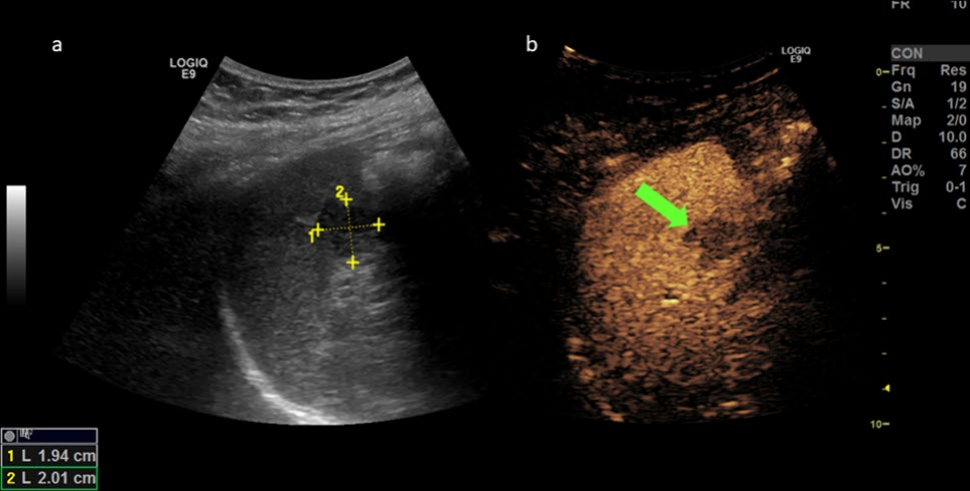
Ultrasound (US) scan of the splenic hypoechoic lesion located near the hilum (2a). Fast wash-out in the venous phase at contrast enhanced ultrasound (CEUS) (2b, the arrows show wash-out in venous phase on B-mode and CEUS)

Contrast enhanced ultrasound (CEUS) was carried out in a supine position to localize the splenic metastasis. The CEUS exam showed two hypoechoic areas near to the splenic hilum, that were characterized by hyperenhancement behavior in the arterial phase followed by rapid washout in the venous phase; those features were suggestive for metastasis (Fig. 1a, b).

Written informed consent was obtained after an extensive office consultation. Institutional review board approval was not requested because this is the anonymous description of a procedure performed in our Ultrasound Unit during clinical practice.

**Fig. 2:**
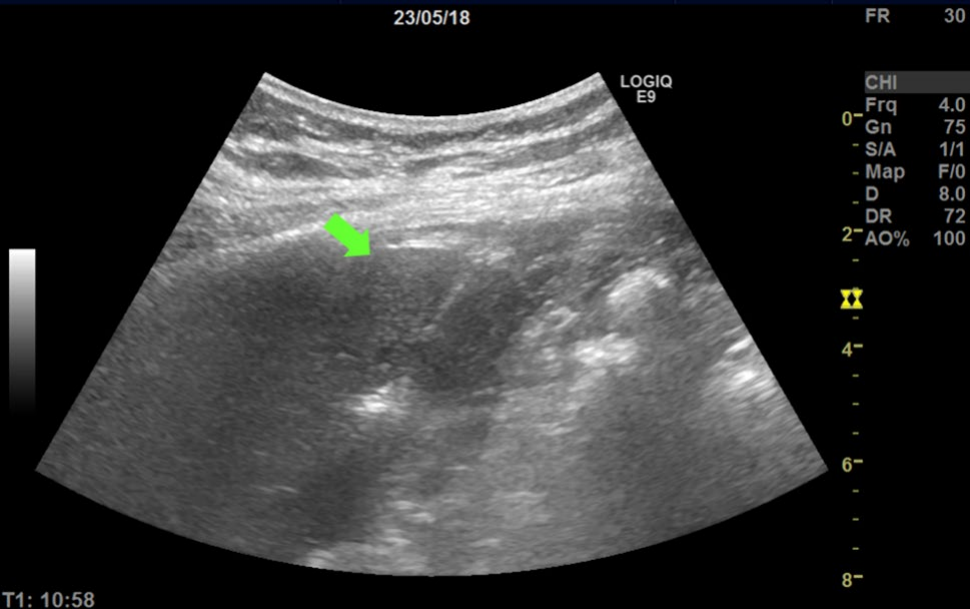
Ultrasound (US) image of the radiofrequency ablation. The image shows a hyperechoic line (needle, pointed with the arrow) inserted in splenic parenchyma

**Fig. 3:**
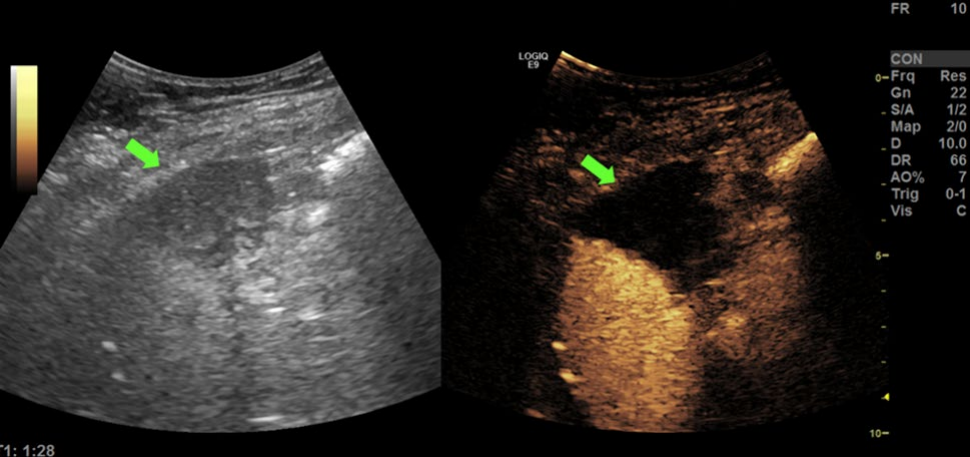
Ultrasound (US) imaging performed the day after the procedure: the image shows the complete necrosis of the treated area (the arrows show the necrosis on B-mode and CEUS)

**Fig. 4:**
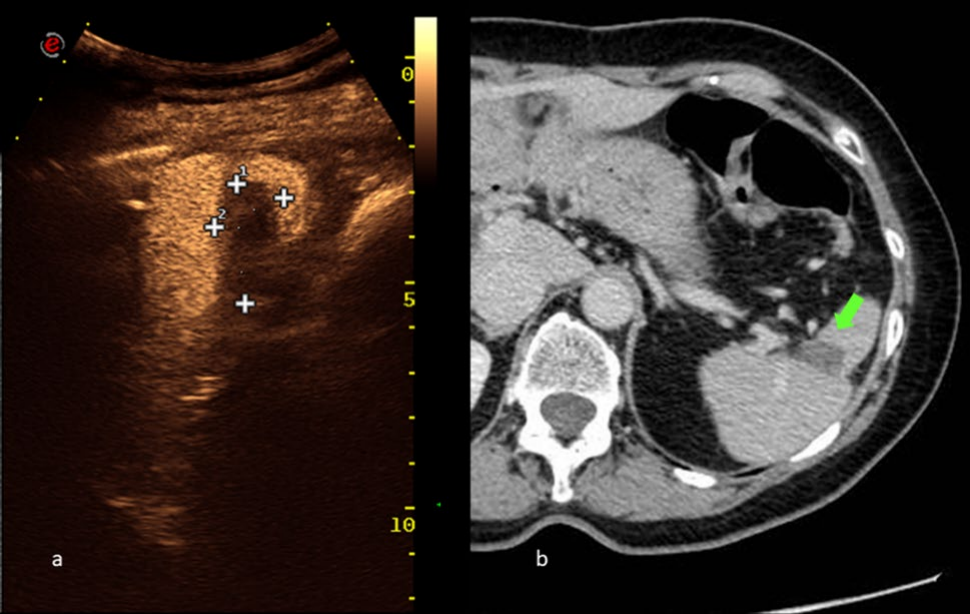
Contrast enhanced ultrasound (CEUS) and computed tomography (CT)-scan imaging at 6-months follow-up. The selected area at CEUS image (5a) shows an anechoic area corresponding to the necrotic area. Late stage-CT scan shows hypodense lesion, corresponding to the lesions treated by radiofrequency ablation in the spleen (pointed with arrow). Both the techniques show no residual tumor

A lateral intercostal approach was used during the RFA treatment with the aim of not passing through the major hilar vasculature and pleura. A cool-tip technique (Radionics generator ablation system) was used. Conscious sedation was administered with intravenous midazolam and intravenous fentanyl with standard hemodynamic monitoring. Tranexamic acid was infused to reduce the risk of bleeding. A 20 cm long needle with a 20 mm non-insulated tip was inserted by using an ultrasound guide (Fig. 2). The two lesions were ablated in the same session requiring 8 min in one and 7 min in the other. The maximum power reached was 118 Watts. After the procedure, the patient was kept overnight for monitoring. The patient reported no pain after the ablation, and the CEUS scan performed the following day showed no complications and complete necrosis of the area treated (Fig. 3). The patient was discharged the day after the procedure. The 6-month follow-up PET/CT and CEUS scans showed no sign of splenic residual disease (Fig. 4a, b).

## Discussion & review of literature

TA is an emerging technique in the local control of metastatic splenic lesions [2]. We described the case of a patient with splenic metastasis from ovarian cancer treated by RFA through a percutaneous approach. To our knowledge, this is the second case of splenic metastasis from ovarian cancer treated percutaneously, the first one treated by RFA. In our case, RFA was carried out with no recurrence of disease at a 6-month follow-up. According to previous data, no major and minor adverse events were reported. Conversely, the morbidity rates for open and laparascopic splenectomies are approximately 27% and 15%, respectively [6].

To our knowledge only 10 cases regarding TA of splenic metastasis have been reported in the literature, for an overall number of 12 metastatic lesions: 6 cases treated with RFA and 4 cases with microwave ablation (MWA) [7–12] (For details, see Table 1). The primary tumors were reported to be melanomas, hepatocellular carcinoma, and colorectal, gastric and ovarian cancer. In the reported works, 5 RFAs were executed surgically (2 laparoscopically and 3 via open surgery), and only one RFA was performed by using a percutaneous approach [7–12]. All of the 5 MW ablations were performed percutaneously [7–12]. No post procedural complications were described in any of the cases [7–12].

**Table 1:** Summary of findings of literature review on works dealing with cases of splenic metastases treated by thermal ablation

	Age	Sex	SM number	Size (cm)	Primary tumor site	Approach	RF or MW	Splenectomy performed	Splenic relapse/persistence	Follow up (months)
Wood et al. [7]	55	M	1	5	Renal cell carcinoma	Percutaneous	RF	No	No	6
Marangio et. al. [8]	60	F	2	1	Colorectal adenocarcinoma	Laparotomy	RF	Yes	Yes (persistence)	
				3						
Yu et al. [9]	55	F	2	1,3	Ovary	Percutaneous	MW	No	Yes	43
				2,8		Percutaneous	MW	No	No	
	56	F	1	1,6	Lung	Percutaneous	MW	No	No	28
	56	M	1	2,5	Stomach	Percutaneous	MW	No	No	4
	32	M	1	2,9	Liver	Percutaneous	MW	No	No	13
Lardière-Deguelte et al. [10]	45	F	1	2,5	Colorectal adenocarcinoma	Laparoscopic	RF	No	No	8
Mudan et al. [11]	60	M	1		Melanoma	Laparotomy	RF	No	No	
Liu et al. [12]	53	M	1	3,4	Liver	Laparoscopic	RF	No	No	8 (died)
	56	F	1	4	Colorectal adenocarcinoma	Laparotomy	RF	No	No	24
The case in the present study	57	F	1	1,3	Ovary	Percutaneous	RF	No	No	4

Abbreviations: *SM* splenic metastasis, *RF* radiofrequency, *W* microwave

In one patient, two splenic lesions were treated at the same time. In one case of ovarian cancer, a splenic relapse occurred 11 months after the MWA, and it was treated by another MWA.

In a case report by Marangio et al. [8], a splenectomy was performed immediately after the RFA; macroscopic analysis showed an area of viable tumor surrounding the area treated. This suggested that splenic metastasis could need greater RF energy than other solid tumors treated with TA.

Due to the proximity of the diaphragm and the high vascularization of the spleen, some specific cautions are needed for splenic percutaneous RFA due to the high risk of thermal injury to adjacent organs and severe bleeding. Therefore, accurate thermal monitoring and a continuous real time ultrasound evaluation are strongly suggested to reduce the risk of overheating injuries and of organ damage, respectively.

Moreover, artificial pleural effusion can be induced when treating upper pole spleen lesions to prevent pulmonary complications [10, 11].

## Conclusion

This case is worth noting since spleen metastasis from ovarian cancer are rare, and percutaneous RFA treatment has not previously been reported. Local thermal ablation may be curative or it could reduce the tumor burden, thereby avoiding surgical therapy and the need for additional systemic therapy, depending on the type of cancer. RFA must be considered in patients with previous surgery, recurrence of the disease and high risk of future recurrence, even in a multiorgan palliative care setting. Therefore, RFA of splenic metastasis seems to be feasible and safe, thus allowing the preservation of the spleen immune function with optimal local control of the neoplasm and a low morbidity rate. The role of this technique, however, has still to be defined, and additional studies are required to evaluate the outcomes, to determinate proper guidelines and inclusion/exclusion criteria, and to define whether the percutaneous approach is preferable or not.

## Data Availability

Data are available from authors on request.
